# Understanding Local Crystallography in Solar Cell Absorbers with Scanning Electron Diffraction

**DOI:** 10.1002/smtd.202501334

**Published:** 2025-09-25

**Authors:** Andrea Griesi, Yurii P. Ivanov, Simon M. Fairclough, Arivazhagan Valluvar Oli, Gunnar Kusch, Rachel A. Oliver, Paola De Padova, Carlo Ottaviani, Udari Wijesinghe, Susanne Siebentritt, Aldo Di Carlo, Oliver S. Hutter, Giulia Longo, Giorgio Divitini

**Affiliations:** ^1^ Electron Spectroscopy and Nanoscopy Istituto Italiano di Tecnologia Via Morego 30 Genova 16163 Italy; ^2^ Department of Materials Science and Metallurgy University of Cambridge Cambridge CB3 0FS UK; ^3^ Laboratory for Photovoltaics Department of Physics and Materials Science University of Luxembourg 41 rue du Brill Belvaux L‐4422 Luxembourg; ^4^ Consiglio Nazionale delle Ricerche Istituto di Struttura della Materia Via Fosso del Cavaliere, 100 Roma 00133 Italy; ^5^ Department of Mathematics Physics and Electrical Engineering Northumbria University Newcastle‐upon‐Tyne NE1 8QH UK; ^6^ Universidad Politécnica de Valencia Instituto de Diseño y Fabricación Camino de Vera s/n Valencia 46022 Spain

**Keywords:** 4DSTEM, CIGS, halide perovskite, machine‐learning, photovoltaics, Sb_2_Se_3_

## Abstract

In thin film photovoltaic devices, the control of grain structure and local crystallography are fundamental for high power conversion efficiency and reliable long‐term operation. Structural defects, grain boundaries, and unwanted phases can stem from compositional inhomogeneities or from specific synthesis parameters, and they need to be thoroughly understood and carefully engineered. However, comprehensive studies of the crystallographic properties of complex systems, including different phases and/or a large number of grains, are often prohibitively challenging. Here, the use of 4D Scanning Transmission Electron Microscopy (4D‐STEM) is demonstrated on cross‐sections to unravel the nanoscale properties of three different materials for photovoltaics: Cu(In,Ga)S_2_, halide perovskite, and Sb_2_Se_3_. These materials are chosen because of the variety of challenges they present: the presence of multiple phases and complex stoichiometry, electron beam sensitivity, and very high density of grains. 4D‐STEM provides comprehensive insights into crystallinity and microstructure, but navigating its large datasets and extracting actionable, statistically sound information requires advanced algorithms. How unsupervised machine learning, including dimensionality reduction and hierarchical clustering, can extract key information from 4D‐STEM datasets is demonstrated. The analytical framework follows FAIR principles, employing open‐source software and enabling data sharing.

## Graphical abstract

The local crystallographic properties play a major role in determining the macroscopic behavior of optoelectronic devices. Here, an approach employing scanning electron diffraction is demonstrated for three materials representative of different thin film solar cell technologies, where electron microscopy is combined with advanced data processing techniques (multivariate analysis, clustering) to extract materials insights.

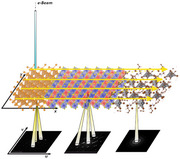

## 1 Introduction

The development of high‐efficiency photovoltaic devices requires detailed microstructural and compositional characterization. Transmission electron microscopy (TEM) is an essential technique for probing the internal structure of these complex materials at the nanoscale. While bulk methods provide information at device scale, the complexity of the phenomena behind operation and their intrinsic length scale requires chemical and structural information at the nm scale to be unraveled. Spurious phases, degradation onsets, and specific grain boundaries need to be identified and understood; this insight can provide input for models and connecting synthesis processes to performance. Focused ion beam (FIB) milling is the most established technique for site‐specific preparation of lamella samples for TEM investigation, enabling precise cross‐sectional analysis of targeted regions. This is particularly important for photovoltaic (PV) materials like copper indium gallium disulfide (Cu(In,Ga)S_2_, CIGS),^[^ ^1^, ^2^, ^3^ ^]^ halide perovskite,^[^ ^4^, ^5^, ^6^ ^]^ and antimony selenide Sb_2_Se_3_,^[^ ^7^, ^8^, ^9^ ^]^ where performance is strongly influenced by grain boundaries, elemental distribution, and interface quality, as they are correlated with charge carrier mobility^[^ ^7^ ^]^ and non‐radiative recombination rates.^[^ ^10^, ^11^, ^12^, ^13^, ^14^, ^15^ ^]^ FIB sample preparation generally introduces surface damage, local heating and ion implantation, and depending on protocols and device architecture, it can affect local composition and crystallinity. However, careful selection of parameters and experimental design, often including comparison of investigated and reference samples, can lead to valuable insight into original morphology, composition, crystallinity, and even optoelectronic properties.^[^ ^16^ ^]^ Although STEM is frequently used to study morphology and composition in solar cell materials, its application to crystallographic analysis is less common. 4D Scanning Transmission Electron Microscopy (4D‐STEM) is a powerful tool for investigating these properties and is highly complementary to traditional TEM approaches, which can sometimes be carried out in parallel. Unlike the more established selected area electron diffraction approach, this technique enables the collection of crystallographic data with nm‐scale resolution over micron‐scale areas. In 4D‐STEM, a focused electron probe is scanned across a sample, and at each position (*x,y*), a diffraction pattern, ***s*** *(u,v)*, is recorded. This process results in a comprehensive 4D dataset, ***I*** (*x,y,u,v)*, that maps real space coordinates to reciprocal space information. The simultaneous acquisition of real‐space *(x,y)* and reciprocal‐space *(u,v)* information in 4D‐STEM generates microscopic maps and diffraction patterns, leading to datasets that can easily exceed 15 GB each. Consequently, statistical and computational approaches are essential for extracting representative information from the entire area and across all layers of a lamella. A variety of statistical and machine learning (ML) techniques,^[^ ^17^ ^]^ such as non‐negative matrix factorization (NMF)^[^ ^18^ ^]^ are available for denoising, dimensionality reduction, and feature extraction from these large datasets.^[^ ^19^ ^]^ Several open‐source software packages are available for applying these statistical algorithms to 4D‐STEM datasets, many of which are based on Python, such as Pyxem^[^ ^20^ ^]^ and py4DSTEM.^[^ ^21^ ^]^

In this study, we present a methodology for extracting key information from large 4D‐STEM datasets recorded on FIB lamellae of absorbers for photovoltaic applications, using unsupervised machine learning techniques, including dimensionality reduction and hierarchical clustering. We discuss how the insight gained from 4D‐STEM can address physics and engineering questions at the nanoscale and support the development of opto‐ and micro‐electronic devices. Examples include identification of crystallographic relationships at grain boundaries, presence of spurious phases in selected grains or interfaces (or lack thereof), and commonalities in orientation for ensembles of grains. To ensure compliance with best data analysis and sharing practices, we follow FAIR (**F**indability, **A**ccessibility, **I**nteroperability, and **R**euse) principles,^[^ ^22^ ^]^ and only employ open‐source, Python‐based software. Furthermore, we suggest an approach for addressing potential problems encountered during the processing stage due to sub‐optimal data collection using a realistic scenario.

## 2 Results and Discussion

### 2.1 Sulphide Chalcopyrite–Cu(In,Ga)S_2_ (CIGS)

CIGS is a promising candidate for thin film photovoltaics, demonstrating power conversion efficiency over 15%^[^ ^2^, ^13^, ^23^ ^]^ in single junction and with a strong potential for specific applications, such as tandem configurations, both ground‐ and space‐based.^[^ ^24^ ^]^ As the Cu‐In‐Ga‐S system has a number of possible stoichiometries and crystal phases,^[^ ^25^ ^]^ the optimization of CIGS devices includes fine‐tuning of local composition, grain size, and phase.^[^ ^26^ ^]^ The efficiency of CIGS solar cells is critically influenced by crystallographic grain structure, with grain size and defects in the bulk being the main factors affecting carrier recombination dynamics.^[^ ^27^ ^]^ Larger grains reduce grain boundary density, minimizing defect‐mediated recombination and enhancing carrier collection efficiency. Different studies demonstrate that absorbers with large grain sizes achieve high power conversion efficiencies, attributed to improved open‐circuit voltage (V_oc_) and fill factor (FF) from reduced boundary losses.^[^ ^28^, ^29^ ^]^ Optimizing CIGS performance thus hinges on balancing deposition conditions (temperature,^[^ ^30^ ^]^ substrate composition^[^ ^31^ ^]^ and back contact) and defect passivation strategies to maximize grain dimensions while ensuring interfacial stability. This grain‐engineered approach positions CIGS as a versatile high‐efficiency technology for next‐generation solar energy systems.^[^ ^32^ ^]^

**Figure 1:**
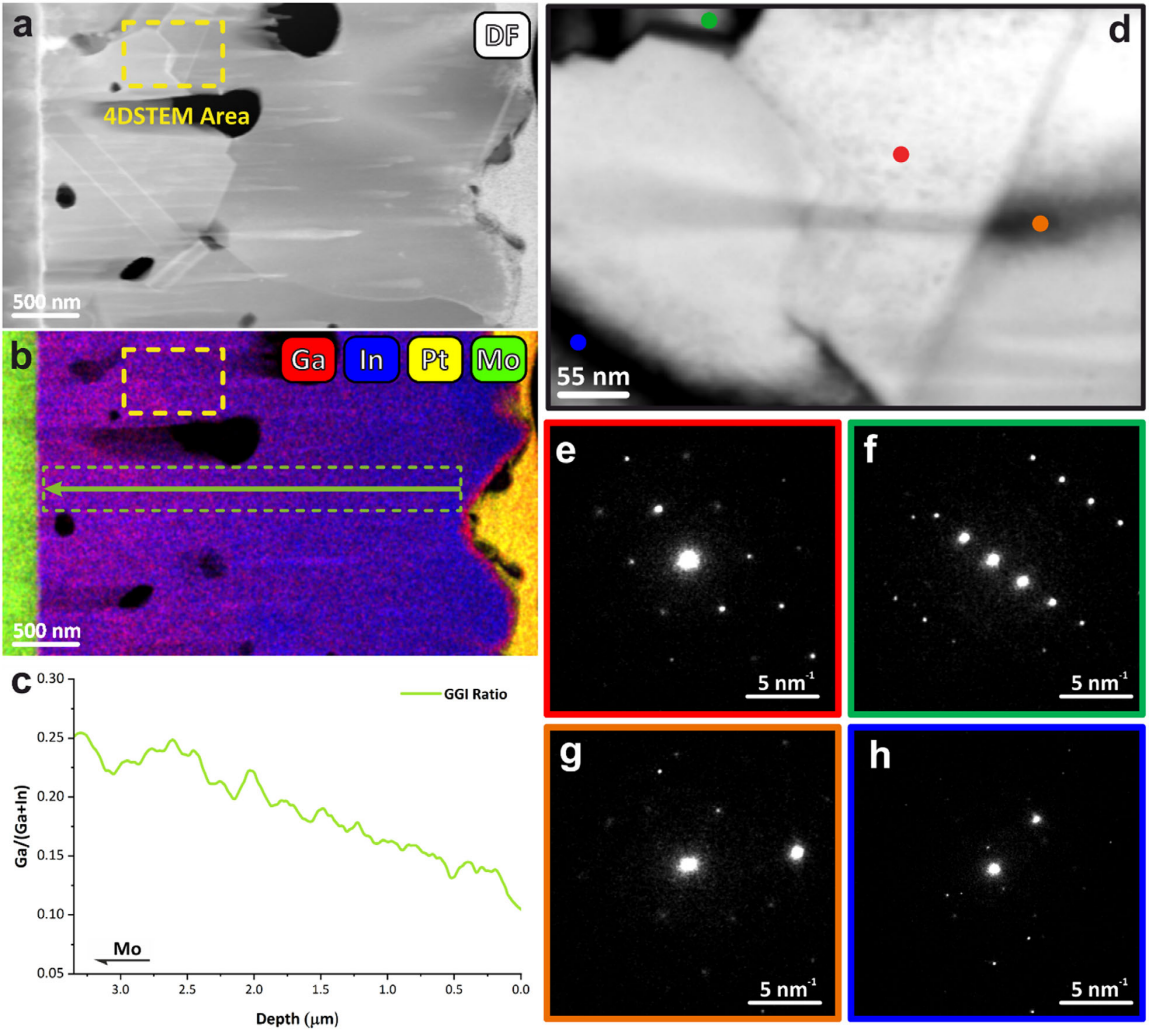
a) Dark field STEM image of a CIGS lamella, where the yellow rectangle indicates the area where the 4D‐STEM data were recorded. b) Composite elemental distribution map of Ga, In, Pt, and Mo from EDX; the green arrow and dashed rectangle denote the linescan region from which the GGI ratio was extracted. c) Depth profile of the GGI ratio through the CIGS layer. d) Virtual bright‐field STEM image of the region highlighted in (a). e–h) Representative diffraction patterns from the corresponding color‐coded positions in d.

4D‐STEM is an ideal technique for quantifying and correlating the degree of crystallinity as a function of the different synthesis and optimization processes for the active layer of CIGS for photovoltaic applications. We employed 4D‐STEM and machine learning to analyse a photovoltaic device that consists of a molybdenum back contact, a chalcopyrite layer exhibiting an intentional compositional gradient, and a platinum protective layer deposited during FIB lamella preparation (complete structure in Figure S1, Supporting Information). In this case, precise tailoring of the fabrication process yields a gradient in the atomic composition, and subsequently a graded bandgap, effectively reducing recombination losses.^[^ ^33^ ^]^ Specifically, optimizing the [Ga/(Ga+In)] (GGI) atomic ratio and its variation through the thickness of the active layer has been shown to improve optoelectronic performance.^[^ ^26^ ^]^ Furthermore, isoelectronic alloying with elements such as silver into chalcopyrite has demonstrated enhanced solar cell performance,^[^ ^2^, ^34^, ^35^, ^36^ ^]^ attributed to increases in grain size and carrier lifetime. This is therefore an excellent example for a TEM investigation to understand the interplay between composition and crystallography at the scale of individual grains. In the sample under analysis, the GGI ratio increases toward the molybdenum contact. In addition, there are small Ga‐rich areas, which can be examined in detail using 4D‐STEM, as can be seen in **Figure** **1**.

Specifically, the use of 4D‐STEM combined with machine learning enables a direct correlation between local crystallography and composition; it becomes possible to verify whether tuning of the GGI ratio and/or the introduction of a dopant atom alters the crystal structure of the active layer. Unlike conventional diffraction or imaging methods, which often provide averaged information across relatively large specimen areas, 4D‐STEM collects a full diffraction pattern at every probe position, thereby generating a 4D dataset that preserves both spatial and reciprocal space information at nanometre resolution. This unique capability allows us to probe local variations in lattice orientation, strain, and symmetry breaking across grains or within a single grain. Consequently, we can identify whether the composition gradient introduces crystallographic strain, promotes the nucleation of small crystalline domains, or modifies the grain boundary structure. Such an approach is particularly powerful for chalcopyrite absorbers, where compositional optimization strategies are known to affect grain size, carrier lifetimes, and ultimately device efficiency.^[^ ^28^, ^29^ ^]^

The first step in navigating 4D‐STEM datasets is the generation of virtual reference images by selecting regions in the diffraction patterns, from which an intensity map can be built. Selecting electrons scattered at high angles, a Virtual High‐Angle Annular Dark Field (VDF) image can be obtained, analogously to what is normally used for navigation in STEM. In this case, the contrast is mainly provided by thickness and composition (average *Z* number). VDF images built with electrons scattered at lower angles or close to/including the transmitted beam (Virtual Bright Field, VBF) also yield information on crystal orientation and other phenomena such as grain boundaries, bend contours, and defects. Therefore, VBF images can be easily used to qualitatively visualize the local crystallography and identify areas for more in‐depth analysis. This step was particularly important due to the presence of large and irregularly shaped domains within the sample, often exacerbated by the presence of pores and artefacts from FIB processing.^[^ ^37^ ^]^ An alternative approach to identifying areas for in‐depth investigation is the use of multivariate analysis: NMF, in particular, is an algorithm particularly well‐suited for analyzing 4D‐STEM datasets, as it effectively handles non‐negative data, such as diffraction intensities, while ensuring physically meaningful results by not further compressing data, exploiting anticorrelations. In particular, the anti‐correlations identified by other dimensionality reduction techniques, such as Principal Component Analysis (PCA), enhance the compression of the dataset. However, this can lead to the loss of physical interpretability, as the resulting principal components may represent mixed or abstract features. NMF, by contrast, does not exploit such anti‐correlations and decomposes the data into additive, non‐negative components that align closely with the physical reality of the dataset. This ensures that the extracted features, such as diffraction intensities, domain‐specific signals, or phase contributions, remain directly interpretable. For instance, in 4D‐STEM datasets, NMF can isolate signals corresponding to distinct grain orientations or phases without introducing artefacts. This property of NMF makes it particularly suitable for 4D‐STEM analysis, where maintaining the fundamental properties of physical signals is critical for understanding material behavior, such as grain boundary characteristics, defect distribution, and crystallographic alignment. By preserving the non‐negative nature of diffraction data, NMF enables a more intuitive and reliable exploration of complex datasets, facilitating targeted investigations into the microstructural features most relevant to material performance. The NMF algorithm simplifies the complexity of a high‐dimensional dataset by representing it in a more compact and interpretable form. Furthermore, NMF tends to generate sparse components, aiding in identifying distinctive physical features, such as different diffraction patterns for different phases. However, NMF's ability to reveal meaningful variations in the data can be compromised by spurious information, such as global shifts in the diffraction pattern caused by imperfect beam shift corrections in the microscope. To obtain crystallographically meaningful results prior to applying the NMF algorithm, several pre‐processing steps were implemented using Pyxem,^[^ ^20^ ^]^ a Python library for electron diffraction data analysis. These simple precautions, explained in Section S2 (Supporting Information), prevent the NMF algorithm from focusing on data collection artefacts and instabilities (such as incorrect relative intensities between reflections in the pattern, detector saturation, etc.), which could generate a large variance in the dataset and hence dominate the decomposition results. Using this approach, we efficiently characterized a large area of the sample within a few minutes, enabling the accurate determination of the number of crystalline grains, including an area of the lamella exhibiting significant surface irregularities. The decomposition suggests that the grain boundaries in this type of sample are not always well‐defined or straight, so they have very different adjacent orientations. The observed contrast variations among the components confirm the presence of a non‐planar surface and variations in sample thickness. Furthermore, the application of the NMF algorithm facilitated the identification of small regions within the sample exhibiting high crystallographic strain. These regions may be of particular interest for optoelectronics due to their potential to exhibit altered electronic band structures and enhanced charge carrier scattering, which can significantly influence device performance.

**Figure 2:**
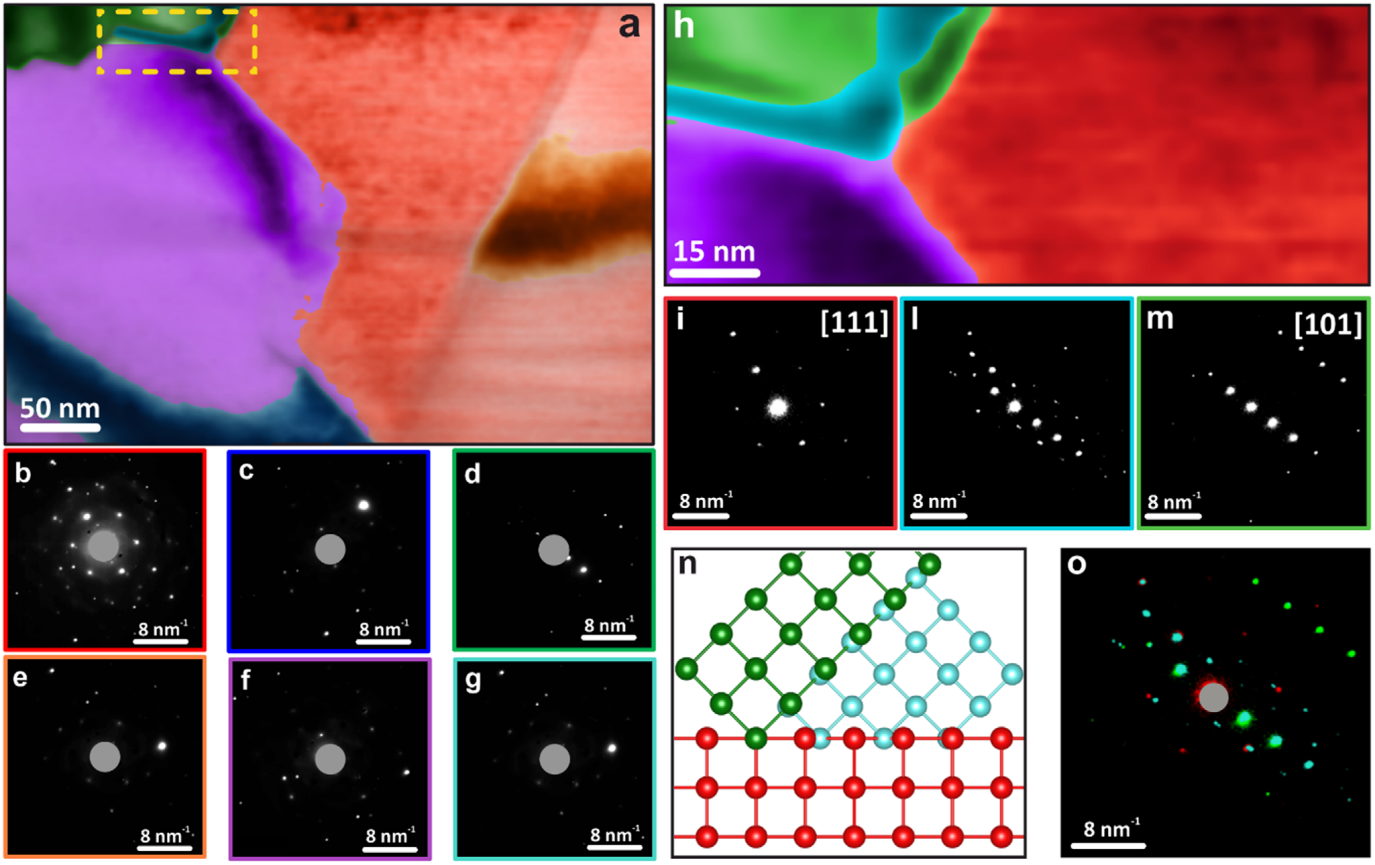
NMF decomposition results on a 4D‐STEM hypermap of a CIGS lamella, overlaid on a VBF image. a) Spatial distribution of the factors b–g) NMF decomposition factors (the central spot has been masked). h) 4D‐STEM NMF decomposition results from the area in the yellow rectangle in (a), with the spatial distribution of the components in chalcopyrite. i–m) Representative diffraction patterns obtained at positions corresponding to the spatial components in (h). n) Reciprocal space orientation relationship between red and green components. o) Superposition of diffraction patterns from the three components (in the same color‐coding).

To confirm the robustness of the NMF decomposition and to rule out the possibility of convergence to a local minimum or the presence of artefacts, we performed another NMF analysis specifically targeted on an area of interest where crystallographic strain might be present. As demonstrated in **Figure** **2**, this analysis yielded consistent results on the entire area. Then, we investigated the relationship between the observed orientations; although different crystal phases can be generally observed in CIGS films, for ease of interpretation we indexed the diffraction patterns using a zinc blende cubic lattice (a = b = c = 5.58 Å, ICSD: 53 293).^[^ ^38^ ^]^ It has been demonstrated that thin films of chalcogenide can present a pseudo‐cubic (tetragonal) crystal structure with a c/a lattice constant ratio with an exceptionally small deviation from unity (linearly varying from ≈0.3% to ≈2% as a function of the GGI ratio),^[^ ^39^ ^]^ so assuming a cubic structure provides an intuitive framework to understand the crystallography of film growth. This analysis revealed that the red component corresponds to a [111] crystalline orientation, while the green component corresponds to a [101] orientation (Figure S2). These findings indicate that the two domains are related by a 45‐degree rotation along one of the <100> directions and that the diffraction pattern in the intermediate region (in light blue) represents a superposition of the diffraction patterns from the two distinct orientations, rather than a crystallographic region with high strain. The indexing of the purple component was not possible due to the small number of reflections present in the diffraction patterns. With this approach, we were thus able to assess the correlation between neighboring crystalline grains. Knowing the relationship between the various crystals within the CIGS film allows us to more effectively optimize the film growth. In particular, the presence of boundaries between two different crystalline orientations has been shown to significantly limit the performance of the device by lowering both the V_oc_ and FF.^[^ ^28^, ^29^ ^]^ This is due to the fact that grain boundaries often act as sites for nonradiative recombination and may introduce defect states that hinder charge carrier mobility. Furthermore, the uneven distribution of electronic levels across these boundaries can create potential barriers that further degrade the efficiency of charge transport. By refining the growth method to minimize the density of such grain boundaries, we can achieve films with more uniform crystalline quality, which is critical for enhancing both the optoelectronic properties of the material and the overall photovoltaic performance.

In this analysis, the combination of 4D‐STEM and ML was instrumental in revealing the nature of grain interfaces, which could be modelled in order to evaluate their effect on charge transport, providing useful information for film engineering. Moreover, the classification of grains confirmed consistency of the crystalline phase through the compositional GGI gradient, excluding the presence of other phases, even in small grains. While other phases could provide non‐radiative recombination sites, they might not be visible in bulk methods like X‐ray diffraction.

### 2.2 Caesium Lead Bromide Perovskite–CsPbBr_3_

Halide perovskites have received significant attention from the scientific community since 2009, following Kojima and colleagues' groundbreaking work on photovoltaic cells based on hybrid organic‐inorganic perovskites.^[^ ^40^ ^]^ This seminal study initiated an intense global research effort, leading to the development of numerous and diverse optoelectronic and PV applications based on this class of materials. Their compositional flexibility, leading to the ability to combine a wide range of elements and compounds, allows the scientific community to design crystals with tailored physical, optical, and electrical properties.^[^ ^41^, ^42^, ^43^, ^44^ ^]^ Among these materials, all‐inorganic caesium lead halide perovskites (CsPbX_3_)^[^ ^45^, ^46^ ^]^ represent a particularly promising class of compounds, primarily due to their enhanced stability and carrier conductivity compared to their hybrid organic‐inorganic counterparts.^[^ ^47^ ^]^ The best photovoltaic performance of CsPbBr_3_ is generally achieved with high crystallinity and minimal presence of non‐radiative recombination centers, often associated with crystal defects and spurious phases.^[^ ^48^, ^49^ ^]^ To achieve high‐quality grain structures, methods like growth in molecular beam epitaxy (MBE) conditions under ultra‐high vacuum (UHV) have emerged, such as one we reported where we grow a layer of CsPbBr_3_ on Si(111) with a nm‐sized amorphous interlayer. This approach provides precise control over the growth conditions, potentially leading to improved crystallinity and compositional homogeneity of the film, thanks to the evaporation of stoichiometric molecules rather than individual species.

To mitigate the damage of the highly beam‐sensitive CsPbBr_3_ perovskite during the recording of 4D‐STEM data, the electron probe employed during the diffraction acquisition was kept at a relatively large diameter, on the order of a few nm, to reduce the electron dose per unit area on the specimen. This choice ensured that a sufficient diffraction signal could be collected while avoiding excessive localization of the beam, which would otherwise result in rapid structural degradation of the perovskite structure. To further prevent beam damage, the acquisition strategy relied on minimizing both exposure time and beam current, combined with a sequential sampling practice that avoided repeated illumination of neighboring regions. The sampling points were spaced at a distance large enough to prevent overlap of the irradiated volumes, thereby preserving the structural integrity of adjacent crystalline domains. In addition, the microscope alignment and focusing procedures were performed in regions outside the area of interest, ensuring that the actual probed sites experienced only the minimum necessary electron exposure. These combined precautions allowed us to record reliable diffraction patterns while maintaining the pristine structure of the material at neighboring sampling points, a critical requirement when working with perovskites of such high electron sensitivity.

We conducted 4D‐STEM measurements to assess the local crystallinity and demonstrate the viability of this approach for halide perovskites, which in principle could be damaged by FIB milling, electron beam damage during FIB sample preparation, and electron beam damage during TEM analysis. In this instance, the CsPbBr_3_ thin film exhibited excellent crystalline quality, with grains having a lateral size comparable to the film thickness. There are no compositional gradients, nor variations in stoichiometry at grain boundaries, as clearly visible from the EDX data (Figure S3, Supporting Information); diffraction data were collected on the entire perovskite layer, including the Si layer on which the perovskite was grown, and the platinum layer deposited to protect the perovskite during the fabrication of the FIB lamella.

**Figure 3:**
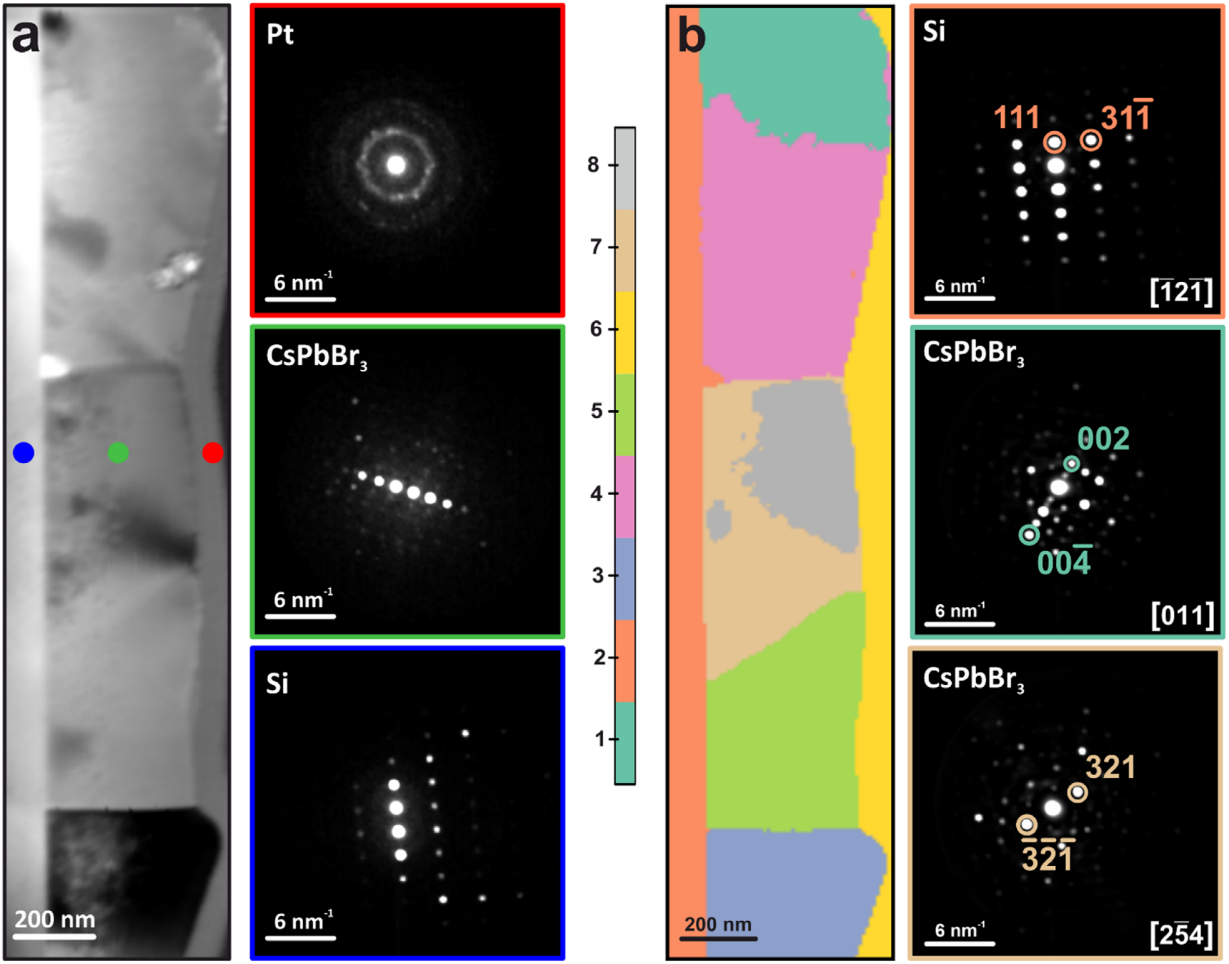
a) Virtual bright‐field STEM image of a perovskite film and representative diffraction patterns obtained at the corresponding colour‐coded positions on the left: the highly polycrystalline Pt layer, the single crystal perovskite, and the single crystal Si substrate. b) Result of clustering on CsPbBr_3_ 4D‐STEM data, and corresponding indexed diffraction patterns.

This dataset presents different challenges compared to the CIGS example. Furthermore, we opted to include a common artefact, the shadow of the high‐angle annular dark field (HAADF) detector, which is often present in 4D‐STEM datasets (Figure S4, Supporting Information); we also operated at a relatively low camera length even if the diffraction spots are closer than in the case of CIGS, in order to maximize the signal to noise ratio, at the cost of smaller spacing between diffraction features. This dataset serves as an excellent case where machine learning (ML) proves effective even under non‐ideal conditions, reflecting real‐world scenarios. The application of ML to distinguish different orientations in the perovskite crystal was more challenging for the algorithms used: the diffraction patterns show very similar intensities for all reflections, they are in close proximity due to the low camera length, and the extinction rules are often violated due to dynamic effects (as the sample is not ideally thin). To facilitate the interpretation of this dataset, we employed ML techniques that can distinguish between different orientations of the perovskite crystals. Specifically, we employed a semi‐automated clustering approach based on unsupervised learning to process the acquired 4D‐STEM data. This method involves providing the entire dataset to the algorithm, which then autonomously classifies and groups individual diffraction patterns based on their inherent characteristics without requiring any a priori knowledge or training (**Figure** **3**).^[^ ^50^ ^]^

Clustering analysis was used as the primary method for identifying distinct crystalline orientations, effectively partitioning the 4D‐STEM dataset into separate groups (clusters) corresponding to different crystallographic directions or phases. In our statistical analysis, we implemented the K‐Means algorithm, a widely used clustering technique that aims to partition the data into *n* groups of equal variance while minimizing inertia. Inertia, in this context, is a measure of the internal coherence or compactness of the clusters, with lower inertia values indicating more tightly grouped and internally consistent clusters. By minimizing inertia, the K‐Means algorithm effectively identifies the most representative crystalline orientations present within the sample (for more information on the algorithm, see Section S2.4, Supporting Information).

The 4D‐STEM data is thus clearly classified into several clusters, corresponding to different crystalline domains that do not possess the same crystallographic relationship with the substrate. Through 4D‐STEM clustering analysis, it is revealed that the perovskite layer comprises several domains, with the *c*‐axis being generally aligned with the normal to the substrate. Eight distinct crystallographic regions are found. Zones 2 and 6 correspond to the silicon substrate (imaged close to the [111] direction) and the amorphous platinum layer used to protect the perovskite layer during FIB‐SEM lamella preparation, respectively. Zones 1, 3, 4, and 5 are oriented close to the [100] zone axis, making them perpendicular to the [001] growth direction of the perovskite. However, the diffraction patterns from zones 7 and 8 show a [2‐54] orientation, which is nonperpendicular to the *c*‐axis, indicating that occasionally crystals might grow in a different orientation, while still achieving a high crystal quality. We ascribe the high crystallinity of the perovskite film to the relaxation of the interfacial stress with the Si substrate due to a thin amorphous layer, as reported in the literature. In this case, as we focus on the overall grain structure of the film, we use a sampling that is too coarse to capture such a layer.^[^ ^51^ ^]^ The K‐Means approach proved to be very robust and suited to analyzing this dataset compared to other ML‐based algorithms: over large areas, lamellae often present some degree of bending/distortion, and diffraction patterns from different positions in a single large grain might be slightly different. Moreover, strong dynamical diffraction effects are sometimes present, which complicate the direct identification of regions with a common crystallographic orientation. Precessing the electron beam is an approach that mitigates these issues, at the cost of additional hardware and alignments; in this case, we point out how post‐processing can also be effective. In particular, while NMF would highlight these differences, K‐Means instead classifies the grains in a more robust fashion, thanks to the inertia implemented in the algorithm.^[^ ^19^ ^]^ Therefore, we have demonstrated that applying machine learning techniques to analyze diffraction data from perovskite film growth can significantly enhance our understanding of the relationship between fabrication methods and material quality. Specifically, the use of molecular beam epitaxy (MBE) under ultra‐high vacuum (UHV) conditions has been shown to facilitate the growth of highly crystalline perovskite films with minimal defects. This precise control over the growth environment reduces the formation of grain boundaries and non‐radiative recombination centers, which are known to adversely affect charge carrier lifetimes and, consequently, the short‐circuit current and overall power conversion efficiency of photovoltaic devices.^[^ ^52^ ^]^

The 4D‐STEM analysis demonstrates here significant improvement over conventional studies of grains in halide perovskites. While a reduction in grain boundary density could in principle be inferred from grain size measurements obtained by conventional scanning electron microscopy (SEM), those studies often suffer from surface effects, require a high electron dose (for example when employing electron back‐scattered diffraction) and have limited spatial resolution, insufficient to resolve grains or sub‐grains on the 10 nm scale, which have sometimes been observed. The reported study not only shows the reciprocal crystal orientations of the grains, but also allows to exclude the presence of spurious phases, for example at grain boundaries, which are often associated with accelerated material degradation. Moreover, sub‐grain structures are clearly isolated and identified, such as the bending in grain 7/8 above.

### 2.3 Antimony Selenide – Sb_2_Se_3_

Antimony‐based chalcogenides are attracting significant interest for PV applications due to their promising optoelectronic properties. Among these, Sb_2_Se_3_ has recently emerged as a compelling, environmentally‐friendly alternative to CdTe in thin‐film solar cells,^[^ ^53^ ^]^ owing to its near‐ideal optical bandgap (1.0–1.2 eV) and high absorption coefficient (>10^5^ cm^−1^ at short wavelengths).^[^ ^15^, ^54^ ^]^ A distinctive characteristic of the Sb_2_Se_3_ crystal structure, highlighted by several studies, is the presence of 1D [Sb_4_Se_6_]_n_ chains. When these chains are aligned perpendicular to the substrate, the density of dangling bonds is reduced, resulting in intrinsically “benign” grain boundaries.^[^ ^15^, ^55^, ^56^ ^]^ Conversely, aligning ribbons in the [001] direction has been shown to minimize non‐radiative recombination losses, further enhancing the appeal of polycrystalline Sb_2_Se_3_ thin films for PV applications.^[^ ^57^ ^]^ This peculiarity of Sb_2_Se_3_ is in contrast with most common inorganic absorbers (e.g., CIGS and halide perovskites), where broken covalent bonds at grain boundaries introduce defect states and recombination centers that impede charge collection.^[^ ^58^ ^]^ In our study, we therefore used 4D‐STEM to verify the degree of crystallinity and the arrangement of the [Sb_4_Se_6_]_n_ units. Sb_2_Se_3_ films were deposited on titanium oxide (TiO_2_) and underwent a thermal post‐treatment process using Flash Lamp Annealing (FLA). FLA, also known as photonic sintering or photonic curing, utilizes high‐energy xenon lamp flashes to rapidly process the sample in a fraction of a second using a single flash. This method engineers material properties without thermally stressing the underlying substrate, thereby enabling the use of soft/flexible substrates, such as plastics.

**Figure 4:**
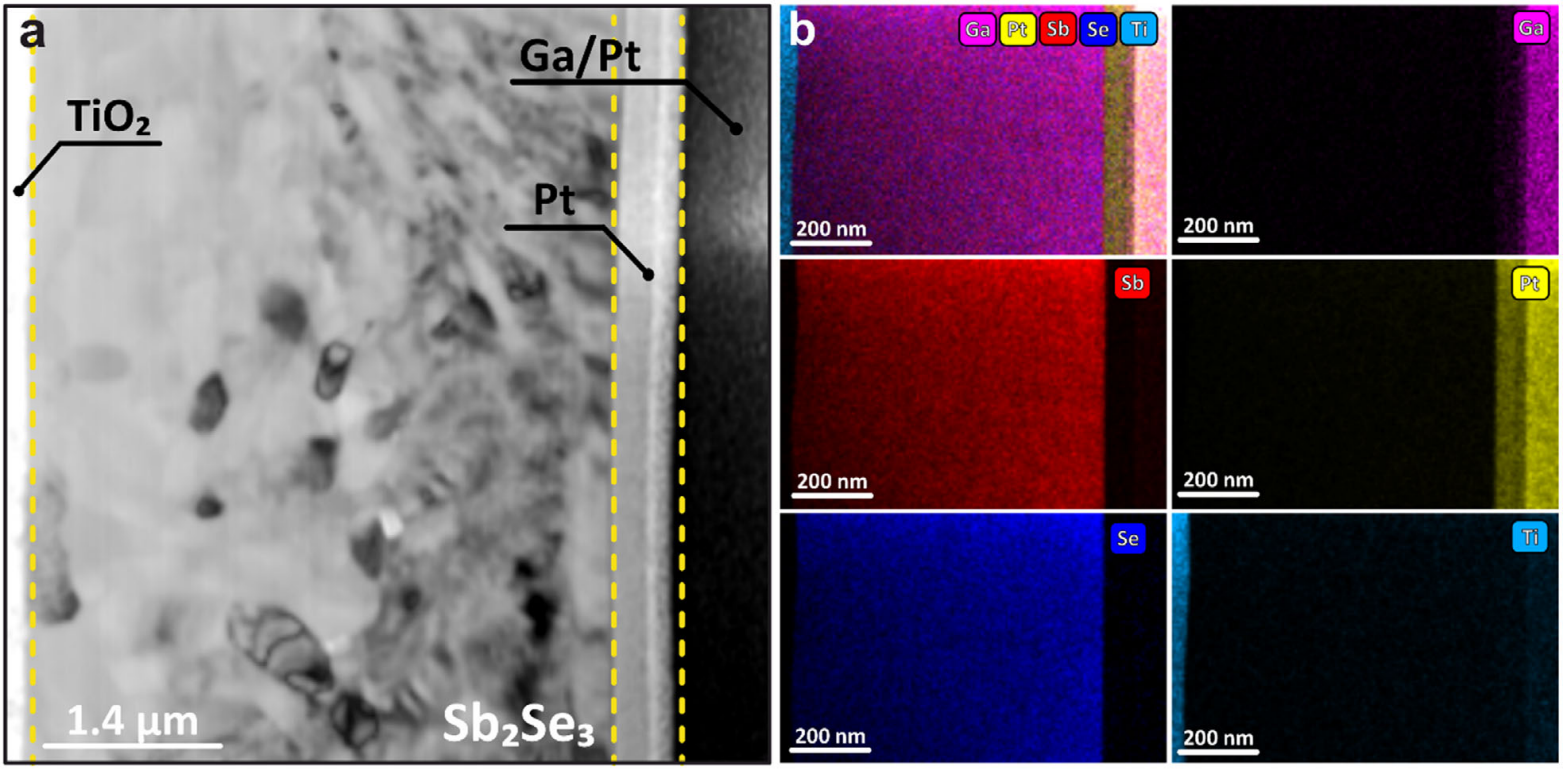
a) Virtual bright field of a Sb_2_Se_3_ lamella from 4D‐STEM data. b) Elemental distribution maps of Ga, Sb, Pt, Se, and Ti from STEM‐EDX.

The EDX elemental maps show no compositional gradients in the chalcogenide layer, indicating a homogeneous composition throughout the film, consistent with the absence of selenium loss observed in film growth or processing.^[^ ^59^ ^]^ All layers are well defined and sharply terminated, and the structure is capped with e‐beam deposited Pt followed by ion‐deposited Pt (**Figure** **4**). The Sb_2_Se_3_ layer is stable under the electron beam (particularly compared to CsPbBr_3_), and we were therefore able to collect a large amount of data with a high resolution. However, this in turn leads to a large, information‐rich dataset containing a larger number of grains compared to the other examples. NMF is the ideal algorithm that can be leveraged to reduce the dimensionality of the data, in order to then apply subsequent clustering algorithms. In this case, we specifically opted to use the Mini Batch K‐Means clustering algorithm, a variant of the classic K‐Means clustering algorithm optimized to handle large datasets. As the size of the dataset increases, the computation time of K‐Means increases due to the need for the entire dataset to be stored in main memory, and an optimized algorithm is thus needed.

**Figure 5:**
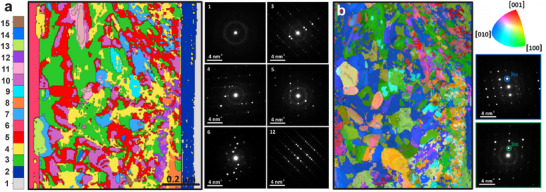
a) Map obtained via the clustering algorithm, and representative patterns for the six obtained components. b) Automated Crystal Orientation Mapping (ACOM) result, where the colors indicate the orientation (relevant map in the top right corner).

The main difference between Mini Batch K‐Means and K‐Means is that the former processes only a small subset of data, called a mini‐batch, in each iteration. The subset is randomly sampled and used to update the obtained clusters, and this process is repeated until convergence. As the number of iterations increases, the effect of new data decreases, and convergence is detected when no changes occur in the clusters over several consecutive iterations. As apparent from **Figure** **5**, the clustering results reveal an increase in the size of the crystal domains as one approaches the titanium oxide layer (component 6). This grain arrangement can be correlated with the fabrication process and potentially determines graded electron transport properties.

Given the high quality of the 4D‐STEM dataset (a large number of reflections for a single pattern, and a high signal/noise ratio), we also conducted Automated Crystal Orientation Mapping (ACOM) using the py4DSTEM library.^[^ ^21^ ^]^ We focused exclusively on the antimony triselenide layer, employing the orthorhombic crystal structure with Pnma(62) symmetry (ICSD: 194836)^[^ ^60^ ^]^ for indexing the patterns. The results are consistent with those obtained from clustering but provide additional insights into the crystal orientation within the layer. However, this approach requires significantly longer computation times for a smaller sample area, with respect to ML methods, as well as a conscious choice of model crystal structure. As can be easily seen, the information from ACOM (Figure 5b) is consistent with the previous approach: there is a significant increase in the average size of the crystals when moving deep into the film (toward the TiO_2_ layer). However, there are subtle differences in the resulting grain classification. The clustering algorithm groups diffractograms into a finite number of clusters, whereas Automated Crystal Orientation Mapping (ACOM) indexes diffraction patterns with a finer granular set of crystalline orientations, thereby using a large number of components to describe the data. This is reflected in the gradual color variation observed in the ACOM map in Figure 5b. The differing approaches of these methods mean that ACOM is inherently more sensitive to variations in orientation, but, unlike clustering, it requires careful optimization of the data, including necessary corrections and calibrations, and lengthy data processing. Thanks to the ACOM and the high quality of the data, we have been able to demonstrate that the Sb_2_Se_3_ layer exhibits [010] and [100] (blue and green in Figure 5) as the most common observed in‐plane directions, especially near the TiO_2_ layer. Given the crystalline structure of the orthorhombic chalcogenides, this growth proceeds in a way that minimizes non‐radiative recombination. This configuration enhances the material's electronic properties, ensuring better charge carrier mobility and reducing energy losses due to recombination along the grain boundaries, which is crucial for improving the performance of optoelectronic devices.^[^ ^57^, ^58^ ^]^

The preferential [010] growth observed in Sb_2_Se_3_ is particularly advantageous because it exploits the intrinsic quasi‐1D ribbon‐like arrangement of covalently bonded (Sb_4_Se_6_)_n_ units aligned along the [001] direction. In this configuration, charge carriers propagate predominantly along the strongly bonded chains, resulting in highly anisotropic transport properties, with lateral transport most efficient along the in‐plane [010] direction rather than [001]. When the crystal grows with the [010] orientation normal to the substrate, the [010] direction lies within the plane of the film, facilitating long‐range lateral carrier transport, while [001] remains the covalent chain axis. Thus, charges can move laterally and find optimal paths to the electrodes, reducing the effects of areas with poor conductivity. Furthermore, the specific nature of Sb_2_Se_3_ grain boundaries has been reported not to be an important problem compared to those in other thin‐film semiconductors, precisely because the continuity of the quasi‐1D chains across grain interfaces can be partially preserved.^[^ ^57^ ^]^ This structural feature mitigates the detrimental impact of grain boundaries on device performance and further explains why the observed orientation is associated with reduced non‐radiative losses. Ultimately, the alignment of the (Sb_4_Se_6_)_n_ ribbons in this favorable configuration provides both efficient charge transport pathways and tolerance to grain boundaries, thus directly linking the growth orientation to the enhanced optoelectronic properties of the material.^[^ ^57^, ^58^ ^]^ 4D‐STEM, here, carried out on a large ensemble of grains, enables the emergence of this finding without introducing potential operator bias.

## 3 Conclusion 

We demonstrated the vast potential for using 4D‐STEM to explore in high detail the nanoscale crystallography of representative emerging solar cell technologies. The use of automated and semi‐automated routines enables the study of samples over large areas and to obtain statistics that can be exploited to optimize fabrication processes. Rather than proceeding through manual analysis of selected areas of the sample being investigated, this workflow provides more solid information. For example, this analysis can confirm that all crystal grains are in the same phase, and includes small features (as the probe size is ∼a few nm) as well as overlapping features (that can be unraveled by ML). Compared to other approaches, such as electron backscattered diffraction, the analyses highlighted here have higher lateral resolution and better dose efficiency. These unsupervised methods can also be applied without introducing operator bias, ensuring an objective estimate of descriptors for each specimen. The deployment of unsupervised computational methods, specifically Non‐negative Matrix Factorization and clustering algorithms, not only expedites data processing but also ensures reliability across a range of materials systems and devices, including beam‐sensitive materials. Furthermore, the use of unsupervised Machine Learning can compensate for the lack of electron beam precession, which would otherwise enhance the accuracy of both diffraction intensities and their spatial distribution during data acquisition. These approaches maximize the extraction of actionable insights from complex electron diffraction datasets, establishing a framework for accelerating the development of next‐generation solar technologies.

## 4 Specimen Preparation

### 4.1 Ag‐Alloyed Sulphide Chalcopyrite–Cu(In,Ga)S_2_ (CIGS)

Ag‐alloyed CIGS absorbers were fabricated using a three‐stage co‐evaporation process, previously established for both sulfide and selenide chalcopyrites.^[^ ^2^, ^13^, ^61^ ^]^ The films were deposited on molybdenum‐coated soda lime glass (SLG) substrates. A 10 nm Ag precursor layer was deposited onto the Mo surface using e‐beam evaporation prior to absorber growth.^[^ ^62^ ^]^ Substrate temperatures (T_s_) were ≈470 °C during the first stage and ≈600 °C during the second and third stages. T_s_ was estimated using a pyrometer calibrated against the substrate heater and the known softening temperature of glass. A ramp rate of 20 °C min^−1^ was used, and substrates were rotated at 8 rpm throughout the deposition. Before the growth process, the Mo/Ag surface underwent sulfurisation in sulfur vapor at T_s_ = 400 °C for 15 min. During the first stage, calibrated fluxes of gallium and indium were co‐evaporated under sulfur partial pressures ranging from 2 × 10^−^⁵ to 8 × 10^−^⁵ mbar. In the second stage, copper was introduced via calibrated evaporation. Upon reaching a (Cu+Ag)‐rich composition with ≈8.5% (Cu+Ag) excess after the stoichiometric point, indicated by an increase in heating power and pyrometer readings, the Cu deposition was stopped, and Ga and In were co‐evaporated again to transition the film to a (Cu+Ag)‐poor composition. In the third stage, the Ga flux was reduced relative to the first stage. The Ag precursor layer served as Ag source during the deposition, facilitating the formation of a (Ag,Cu)(In,Ga)S_2_ bulk absorber with a homogeneous Ag concentration.

### 4.2 CsPbBr_3_

The growth of samples was performed in UHV (≈4.0 10–10 mbar base pressure), using molecular beam epitaxy. The Si(111) substrate was subjected to a thermal treatment in UHV for the removal of carbon, a contaminant originating from hydrocarbons in ambient air, leaving a clean surface terminated with the native SiO_2_. Subsequently, the perovskite growth was performed by exposing the prepared surface at a rate of ≈1–2 Å s^−1^ to CsPbBr_3_ molecules, maintaining the substrate at a temperature of ≈130 °C, for a thickness of ≈300 nm.

The CsPbBr_3_ powder was purchased from TCI, CAS RN:15243‐48‐0, with low water content, without any further treatment.

### 4.3 Sb_2_Se_3_

The Sb_2_Se_3_ films studied here were fabricated following the method reported in Wijesinghe et al.^[^ ^59^ ^]^ FTO‐coated glass (TEC15, Sigma‐Aldrich) substrates were sequentially cleaned in DI water, acetone, and isopropanol using ultrasonic baths, followed by a 15‐min UV‐ozone treatment. A compact TiO_2_ layer was deposited by spin‐coating a 0.30 m titanium isopropoxide solution (97%, Sigma Aldrich) in ethanol twice at 3000 rpm for 30 s, with intermediate drying at 120 °C in N_2_‐filled glove box. The coated substrates were then annealed in air at 450 °C for 30 min and rapidly cooled. Sb_2_Se_3_ thin films were deposited by thermal evaporation (Oerlikon Leybold) at room temperature and immediately treated by photonic curing using a NovaCentrix PulseForge Invent system. Curing was performed under a nitrogen environment at room temperature with a single pulse with a pulse length of 1 ms at 1.55 J cm^−2^.^[^ ^59^ ^]^

## 5 4D‐STEM Experiment

An aberration‐corrected scanning transmission electron microscope (Thermo Fisher Scientific, Spectra 300) was used at an acceleration voltage of 300 kV. The diffraction patterns for the 4D‐STEM data were acquired using a CMOS camera (Gatan Continuum camera). The beam was set up in an almost‐parallel configuration (semi‐convergence angle ≈0.5 mrad) using a 10 µm condenser aperture.

**Table:** 

	CIGS	CsPbBr_3_	Sb_2_Se_3_
Acceleration voltage	300 kV	300 kV	300 kV
Pixel in Real Space	182 × 123	89 × 540	127 × 56
Area in real space	0.728 × 0.492 um^2^	0.445 × 2.74 um^2^	1651 × 0.733 um^2^
Pixel dwell time	0.01 s	0.0062 s	0.01 s
Total acquisition time	6 min	15 min	9 min
Dose (e^−^ Å^−2^)	78	45	12
Camera length	38 mm	38 mm	81 mm
Pixel size (Reciprocal Space)	0.081 nm^−1^	0.032 nm^−1^	0.075 nm^−1^
4D‐STEM data size (in .hdf5 format)	19 GB	8 GB	12 GB

## Conflict of Interest

The authors declare no conflict of interest.

## Supporting information



Supporting Information

## Data Availability

The data that support the findings of this study are openly available in Zenodo at 10.5281/zenodo.15789295, reference number 15789295.
