# Supplementary material for: Understanding Local Crystallography in Solar Cell Absorbers with Scanning Electron Diffraction
Source: Small Methods. 2025 Sep 25;9(11):e01334. doi: 10.1002/smtd.202501334 (PMC12641347; doi:10.1002/smtd.202501334)
Supplement: Supplementary file 1 — Supporting Information [file SMTD-9-e01334-s001.docx]

Understanding local crystallography in solar cell absorbers with scanning electron diffraction

*Andrea Griesi^*^, Yurii P. Ivanov, Simon Fairclough, Arivazhagan Valluvar Oli, Gunnar Kusch, Rachel A. Oliver, Paola De Padova, Carlo Ottaviani, Udari Wijesinghe, Susanne Siebentritt, Aldo Di Carlo, Oliver S. Hutter, Giulia Longo, Giorgio Divitini^*^*

**1. Supplementary figures**


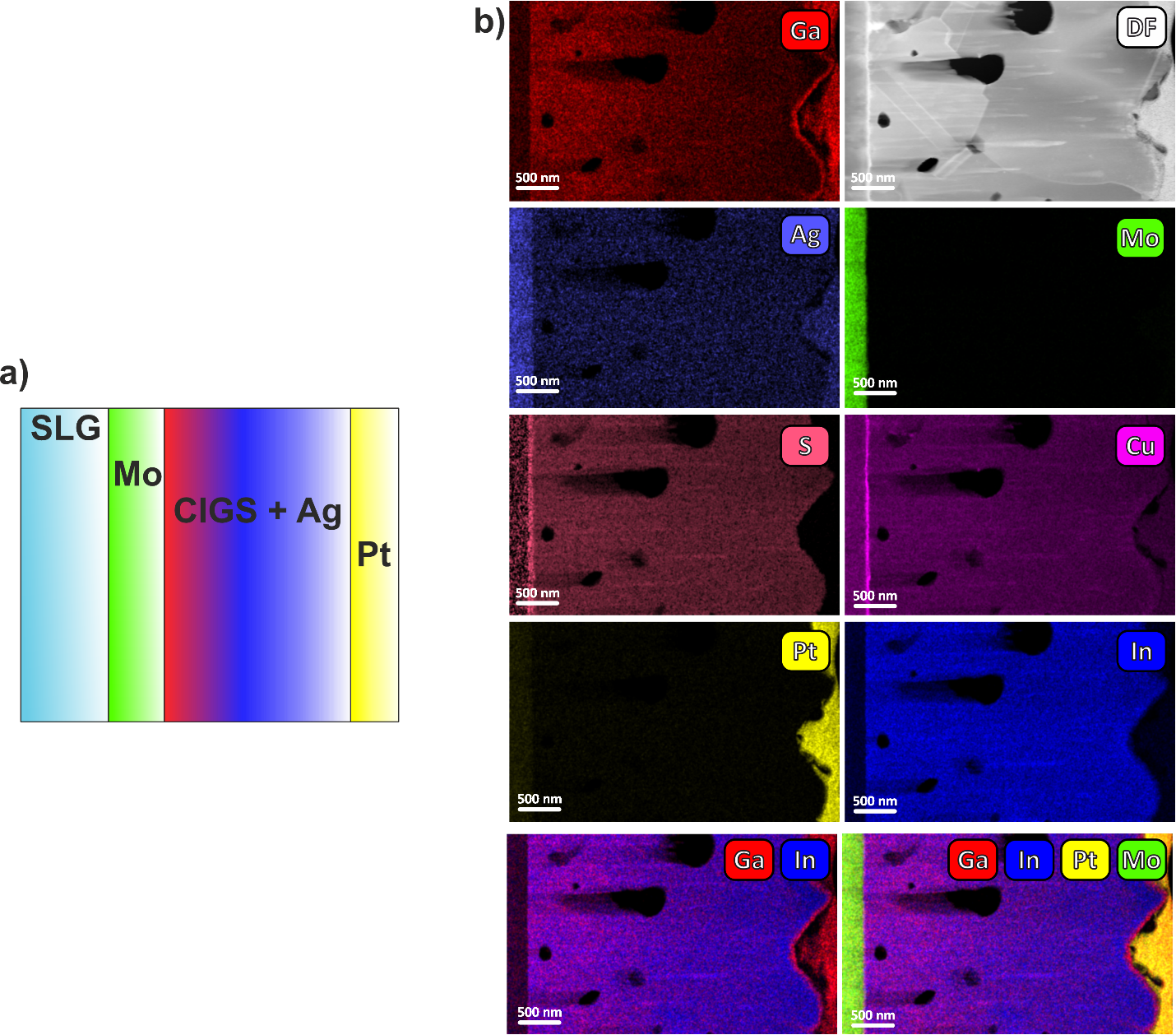


Figure S1: *a) Diagram of a full device. SLG: soda-lime glass. b) Elemental distribution map from STEM-EDX of all components of CIGS as well as the Mo contact and the Pt protective layer.*

*
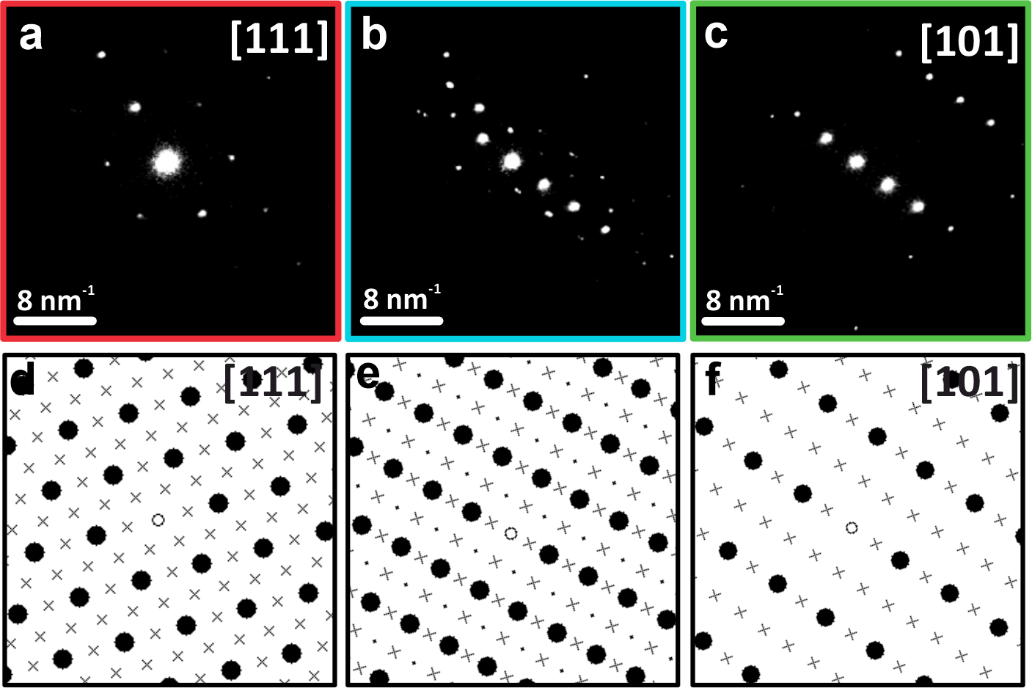
*

*Figure S2: Comparison between the experimental diffraction patterns of the components and the theoretical patterns, calculated with CrysTbox^1^.*

*
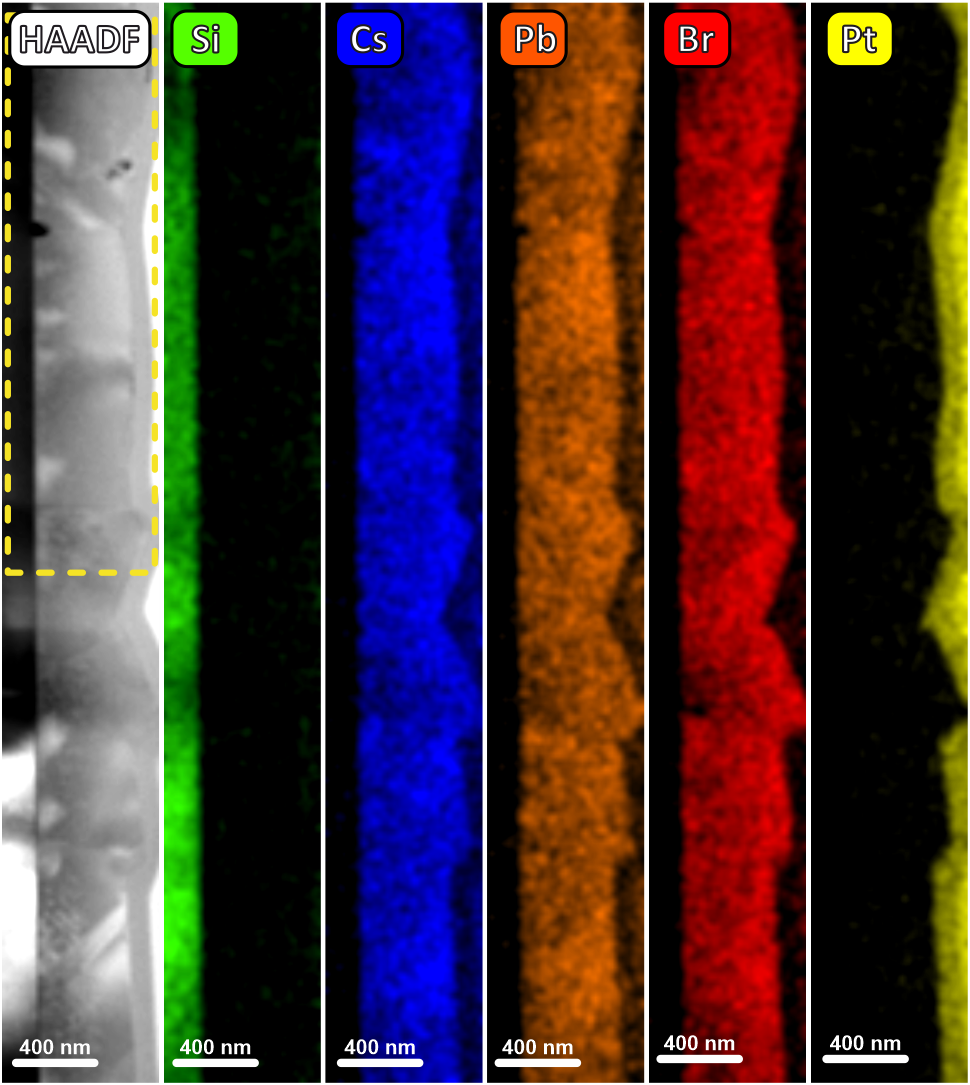
*

*Figure S3: From left: HAADF image of a CsPbBr₃ lamella and elemental distribution maps of Si, Cs, Pb, Br, and Pt. The yellow rectangle indicates the area where the 4D-STEM data were recorded.*

*
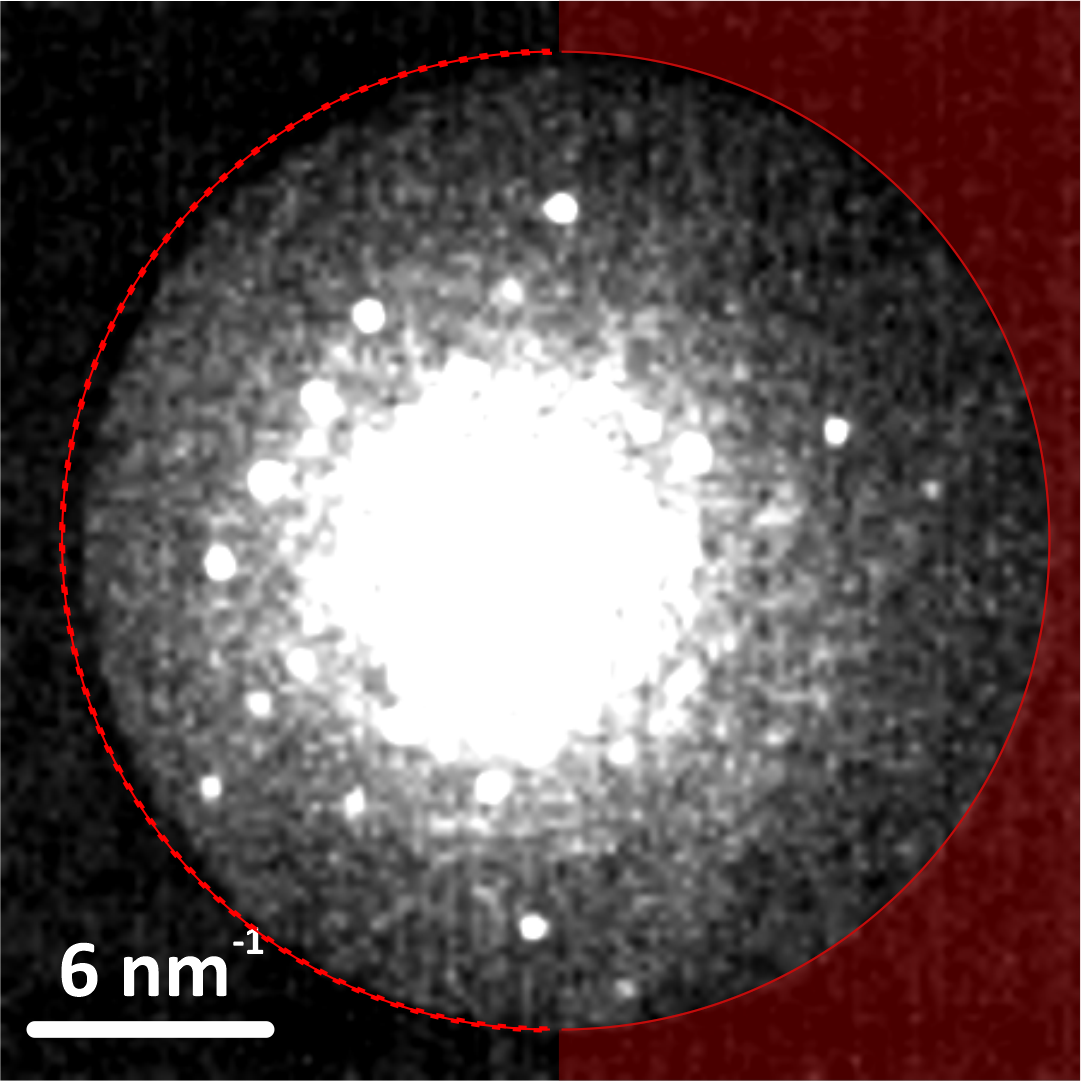
*

*Figure S4: Single diffraction pattern of CsPbBr_3_ with increased contrast (saturated intensity) to make the shadow of the high-angle annular dark field (HAADF) detector visible (in red).*

**2. Methodological background**

**2.1. Non-negative matrix factorization**

In 4D-STEM data acquired from FIB lamellae, and indeed in all 4D-STEM datasets, the measured signal represents a linear combination of essential components, each contributing with a specific weight. Non-negative matrix factorization (NMF) provides an effective method for dimensionality reduction by decomposing the experimental data matrix, **X**, into a product of two matrices: **S**, representing a basis set in reciprocal space of diffraction patterns, and **C**, encoding their corresponding spatial distributions in real space. Thus, the experimental data can be approximated as:

$$X=SC$$

where **C** contains non-negative coefficients, visually interpretable as spatial distribution maps. Defining $n_{x}$, $n_{ky}$ as the number of pixels of the maps in real space, and $n_{u}$, $n_{v}$ as the number of pixels of the diffraction patterns in the reciprocal space, and denoting the total number of components in the dataset as $n_{k} (n_{k}<<n_{xy}$), the dimensions of the matrices are as follows: $\boldsymbol{X\in}\mathbb{R}_{\boldsymbol{+}}^{\boldsymbol{n}_{\boldsymbol{uv}}\boldsymbol{\times}\boldsymbol{n}_{\boldsymbol{xy}}}$, $\boldsymbol{S\in}\mathbb{R}_{\boldsymbol{+}}^{\boldsymbol{n}_{\boldsymbol{uv}}\boldsymbol{\times}\boldsymbol{n}_{\boldsymbol{k}}}$ and $\boldsymbol{C\in}\mathbb{R}_{\boldsymbol{+}}^{\boldsymbol{n}_{\boldsymbol{k}}\boldsymbol{\times}\boldsymbol{n}_{\boldsymbol{xy}}}$ where $n_{xy}= n_{x}\times n_{y}$ and $n_{uv}= n_{u}\times n_{v}$.

To apply NMF, the four-dimensional dataset $I_{4D}(x, y, u, v)$ must be transformed into a two-dimensional matrix **X**. This is achieved by reshaping each diffraction pattern $s(u, v)$ at each real-space position c_k_(*x*, *y*) into a one-dimensional column vector, which then forms a column of the matrix **X**. NMF then decomposes **X** into a matrix **S** containing the basis diffraction patterns in reciprocal space and a matrix **C** containing the corresponding spatial distributions in real space.

The NMF algorithm, as applied to this type of data, generally consists of the following steps:

1. **Selection of the number of components, *n_k_.*** This is the only step where operator input can have an effect. The number of components should be chosen carefully, generally considering the expected complexity of the data (i.e. number of phases or grains).
2. **Initialisation of matrix C.** The elements of the matrix **C** are initialised with non-negative, uniformly distributed random numbers.
3. **Estimation of matrix S.** The matrix **S** is estimated using the following equation:

$$S = {(XC}^{T})- {{(CC}^{T})}^{-1}.$$

4. **Non-negativity and normalisation of S.** Negative values in **S** are set to zero. Each column vector of **S** is then normalised.
5. **Estimation of matrix C.** The matrix **C** is estimated using the following equation:

$$C = {(S^{T}S)}^{-1}-(S^{T}X)$$

6. **Non-negativity of C.** Negative values in **C** are set to zero.
7. **Sorting of components.** The rows of **C** are sorted by their l_2_ norm. The columns of **S** are re-arranged to match the corresponding row order in **C**.
8. **Convergence check.** The mean squared error (MSE) is calculated to evaluate the quality of the approximation: $MSE =-\left( n_{xy} \times n_{uv} \right)^{-1}\sum{(X - SC)}^{2}$. Current MSE is compared to the MSE from the previous iteration; if lower but above convergence threshold, this brings the process back to step 3.
9. **Global minimum search.** To mitigate the risk of converging to a local minimum, steps 2-8 are repeated multiple times, **S** and **C** corresponding to the minimum MSE obtained are selected.

As the number of components, ***n_k_***, increases, the minimum mean squared error (MSE) is generally expected to decrease monotonically. This is because a larger number of components provides a more flexible basis for reconstructing the experimental data. Therefore, the dependence of the minimum MSE on ***n_k_*** can be leveraged to estimate a suitable or plausible value for the number of components. However, it is crucial to avoid overfitting the data by using an excessively large ***n_k_***. Overfitting can lead to the identification of spurious components, which, in the context of crystallographic analysis, may be manifested as an overestimation of the number of crystalline domains within the sample.

**2.2.** **General clustering process**

Clustering is an unsupervised machine-learning technique used to group data into clusters based on their similarity. The goal is to partition a dataset into distinct groups such that data points within the same cluster are more similar to each other than to those in other clusters; this algorithm is perfect for finding the same crystal orientation in 4DSTEM data.

To quantify the similarity between data points, we can define a similarity matrix $A$, which is a symmetric matrix where each element $A_{ij}\geq0$ represents a measure of similarity between the data points indexed by $i$ and $j$. The general approach involves constructing a Laplacian matrix $L$ derived from $A$. Various definitions of the Laplacian exist, each with distinct mathematical interpretations, but they all serve to obtain the connectivity and structure of the dataset.

The algorithm relies on the eigenvectors of the Laplacian matrix. Specifically, we focus on the eigenvectors corresponding to the smallest eigenvalues of $L$, excluding the smallest eigenvalue, which is always zero. These relevant eigenvectors provide a lower-dimensional representation of the data but that preserves its structural properties. For computational efficiency, these eigenvectors are often obtained from a function of the Laplacian that emphasizes larger eigenvalues, allowing for faster convergence and reduced computational complexity.

The algorithm generally consists of the following steps:

1. **Calculation of the Laplacian.** Construction the Laplacian matrix $L$ from the similarity matrix $A$.
   The Laplacian can be defined in various ways, such as the un-normalised Laplacian $L=D-A$, where $D$ is the degree matrix $(D_{ii}=\sum_{j} A_{ij})$.
2. **Compute eigenvectors.** Determination the first $k$ eigenvectors corresponding to the smallest non-zero eigenvalues of $L$*.* These eigenvectors will form a new feature space for clustering.
3. **Feature matrix formation.** Construction a feature matrix using the eigenvectors calculated; specifically, each row of this matrix corresponds to a data point and defines its features in the transformed space.
4. **Cluster nodes.** Application a standard clustering algorithm, such as K-Means, on this new feature matrix to partition the data points into clusters based on their features.

In summary, clustering provides a powerful framework for identifying complex patterns within high-dimensional datasets by leveraging graph theory and linear algebra. Its ability to uncover non-linear relationships makes it particularly useful in various applications.

**2.3. Pre-processing steps with Pyxem**

As first processing steps, the acquired datasets were calibrated against a dataset acquired on a gold cross-grating in the same conditions, with further verification available through measurement of the lattice parameters of the silicon substrate or the platinum capping layer, where present. Cropping of the diffraction patterns to the outermost detectable spot (visible integrating all diffraction patterns) enabled reduction of file sizes and easier computing. Binning of the diffraction patterns can greatly reduce data complexity, however it is best to maintain the original pixel size at least until the central spot is aligned. If the aim is classification, binning can then be carried out to enable large areas at the cost of inaccuracy in spot position; if fine changes in spot position are sought, binning should be avoided. It is generally advisable to test classification and other ML routines on a smaller number of diffraction patterns before and after any binning.

Some features or artefacts from the acquisition process can, in some cases, generate enough variance to be erroneously identified as essential components by the NMF algorithm, leading to overcounting the components of the dataset. These include, for example, diffraction patterns that are misaligned or areas where the shape of the incident beam is distorted due to sample interactions or imperfect instrument alignment. Such mis-identification can introduce components into the decomposition that are not representative of the sample. To avoid these problems, we have first calculated the shift of the incident beam and corrected it by rigidly aligning the diffraction patterns using the central spot as reference. Second, intensities arising from the direct beam incident on the sample were masked – the much higher intensity of the central spot compared to the diffracted beams means that small variations in the intensity of the former greatly overshadow signal from the latter. The last processing step to enhance the physical interpretability of the dataset decomposition involves normalising the intensities of all diffraction patterns relative to the intensity of the brightest reflection observed across the entire dataset. This normalisation procedure offers two advantages. It mitigates the effects of the shapes of reflections, reducing artifacts arising from surface irregularities in the sample, and enables the definition of crystalline domains even when the precise pixel size of the diffraction patterns is not accurately known.

**2.4. K-Means algorithm**

The K-Means algorithm operates by partitioning the dataset into disjoint clusters, $x_{i}$, each characterised by the mean, $\mu_{j}$, of the data points - in this case a mean diffraction pattern - within the cluster. The objective is to identify clusters that minimise the sum of squares within-cluster, known as inertia:

$$\sum_{i=0}^{n} \min_{\mu_{j}\in C} \left( \left\| x_{i}-\mu_{j} \right\|^{2} \right)$$

This approach is particularly effective when spatially-separated domains might contain similar signal, such in the case of distinct, separate grains viewed along the same orientation.

1. M. Klinger and A. Jäger. Crystallographic Tool Box (CrysTBox): automated tools for transmission electron microscopists and crystallographers. Journal of Applied Crystallography, 48(6), 2015. doi:10.1107/S1600576715017252.
